# Case Report: Kawasaki disease associated with acute generalized exanthematous pustulosis secondary to carbocysteine

**DOI:** 10.3389/fped.2024.1374448

**Published:** 2024-03-22

**Authors:** Takashi Furuta, Hiroyuki Fukumoto, Mayu Fujiwara, Shinnosuke Fukunaga, Yuichi Ishikawa, Reiji Hirano

**Affiliations:** Department of Pediatrics, Yamaguchi-ken Shimonseki Saiseikai General Hospital, Yamaguchi, Japan

**Keywords:** acute generalized exanthematous pustulosis, damage-associated molecular patterns, Kawasaki disease, microbe-associated molecular patterns, pathogen-associated molecular patterns

## Abstract

Acute generalized exanthematous pustulosis (AGEP) is an uncommon eruption characterized by sterile pustules on an erythematous background, which is usually associated with drugs. AGEP is described as a self-limiting disease with favorable prognosis. We reported a case of Kawasaki Disease (KD) following AGEP. A 3-year-old male, who was admitted with pustules and five days of fever at our hospital, was diagnosed with AGEP. Despite the skin lesions and fever improving drastically after prednisolone therapy, the fever recurred on hospitalization day 5. The following symptoms suggestive of KD also appeared: bulbar conjunctival hyperemia, cervical lymphadenopathy, erythema of the lips, eruption on his trunk, and erythema and edema of the hands and feet. He was diagnosed with KD and treated with intravenous immunoglobulin. He was discharged on the thirteenth day of hospitalization without cardiac complications. Drug-induced lymphocyte stimulation test revealed carbocysteine as the suspected cause of AGEP, which consequently triggered KD. Because a mucosal lesion is uncommon in AGEP, bulbar conjunctival hyperemia suggested that KD sequentially occurred after AGEP. Since AGEP is benign and self-limited in most cases, it is necessary to differentiate other diseases, including KD, when recurrent fever or rash occurs in the course of AGEP.

## Introduction

Acute generalized exanthematous pustulosis (AGEP) is a rare, acute eruption characterized by the development of several non-follicular sterile pustules on an erythematous background (1, 2). AGEP is associated with drug use in approximately 90% of cases (2). Notably, various drugs have been reported as causing AGEP, and antibiotics are the most typical triggers (2). The onset of AGEP reaction post-drug administration can be divided into two groups: one with a rapid onset (a few hours to 2–3 days post-drug intake) and another with a delayed onset (1–3 weeks post-drug intake) (1, 2). AGEP is described as a self-limiting disease with a favorable prognosis. Kawasaki disease (KD) is an acute febrile illness characterized by systemic vasculitis in infants and young children (3). Notably, many microbes or microbe-derived substances are reported to be associated with KD; however, its etiology remains unclear (4). KD can sometimes lead to coronary artery lesions, which range from dilatation to aneurysms (3). Delayed diagnosis and persistent fever are associated with an increased risk of developing coronary artery lesions (3). Furthermore, fever and eruption, diagnostic criteria for KD, are also observed in other diseases, such as infections and drug allergies. Notably, KD can be a self-limiting disease; however, accurate diagnosis and treatment are critical to prevent cardiovascular complications in patients with KD (3).

Herein, we present a rare case of KD following AGEP.

## Case report

A 3-year-old male was admitted with pustules and five days of fever at our hospital. He had no remarkable medical history, including psoriasis and severe acute respiratory syndrome coronavirus 2 (SARS-CoV-2) infection. He had been given a 7-day-course of carbocysteine 7 days prior to admission because of his respiratory symptoms. He developed a fever 5 days before admission and started acetaminophen. In addition, erythematous papules and plaques on the cheek and the groin area appeared 4 days prior to admission, and rapidly extended to the trunk and limbs. Over the next 24 h, pustules developed within them. Carbocysteine was restarted 3 days before admission. Furthermore, cefditoren pivoxil was started simultaneously with carbocysteine, already after the appearance of his rash and systemic symptoms (Supplementary Figure S1).

**Figure 1:**
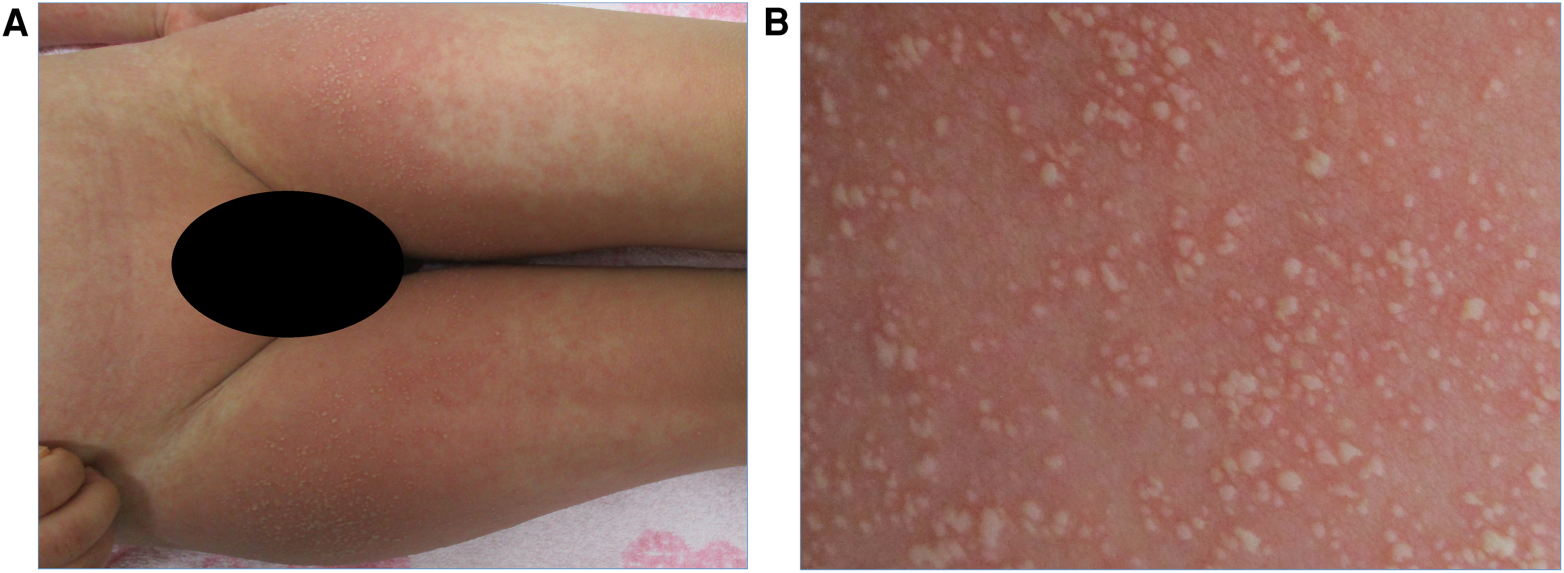
(**A**) Pustular rash with erythematous background on the groin area on admission. (**B**) Close-up view of the same region showing small and non-follicular pustules.

Physical examination revealed the following: body temperature of 37.0°C, heart rate of 122 beats per minute, respiratory rate of 36 breaths per minute, systemic oxygen saturation of 98%, and blood pressure of 111/67 mmHg. No murmurs or crackles were heard on chest examination. A maculopapular rash covering a large area of his body was observed. Notably, erythematous papules coalesced into plaques on the cheek, inner side of the elbows, and the groin area. In addition, a pustular rash with an erythematous background appeared on the flexural areas, including the inner side of the elbows and the groin area (Figures 1A,B). However, no pustular rash was observed on the neck and axilla. There were no mucosal lesions at the time of admission.

Laboratory tests revealed a white blood cell count of 15.7 × 10^9^/L (neutrophils: 41%, lymphocytes: 44.5%, monocytes: 8.0%, eosinophils: 5.0%, basophils: 0.5%), hemoglobin concentration of 10.9 g/dl with a hematocrit of 32.7%, platelet count of 327 × 10^9^/L, and C-reactive protein of 1.68 mg/dl.

The patient's Epstein-Barr Virus and cytomegalovirus serology were negative for both IgG and IgM, suggesting that the patient had never been infected with either virus. Antigen tests for SARS-CoV-2 and influenza virus were negative. Following negative results from skin swabs, blood, and throat swab cultures, no bacterial or fungal pathogens were identified. A skin biopsy was not performed because parental consent was not obtained.

The clinical features, flexural distribution and timing of the eruption and systemic symptoms 10 days after initiation of carbocysteine strongly suggested the diagnosis of AGEP. The patient was treated with intravenous prednisolone (1 mg/kg/day). The fever resolved within a few days, and the pustular eruption showed improvement, followed by desquamation with collarettes of scale. The diagnosis of AGEP was established based on meeting the European Study of SCAR study group criteria (1).

**Figure 2:**
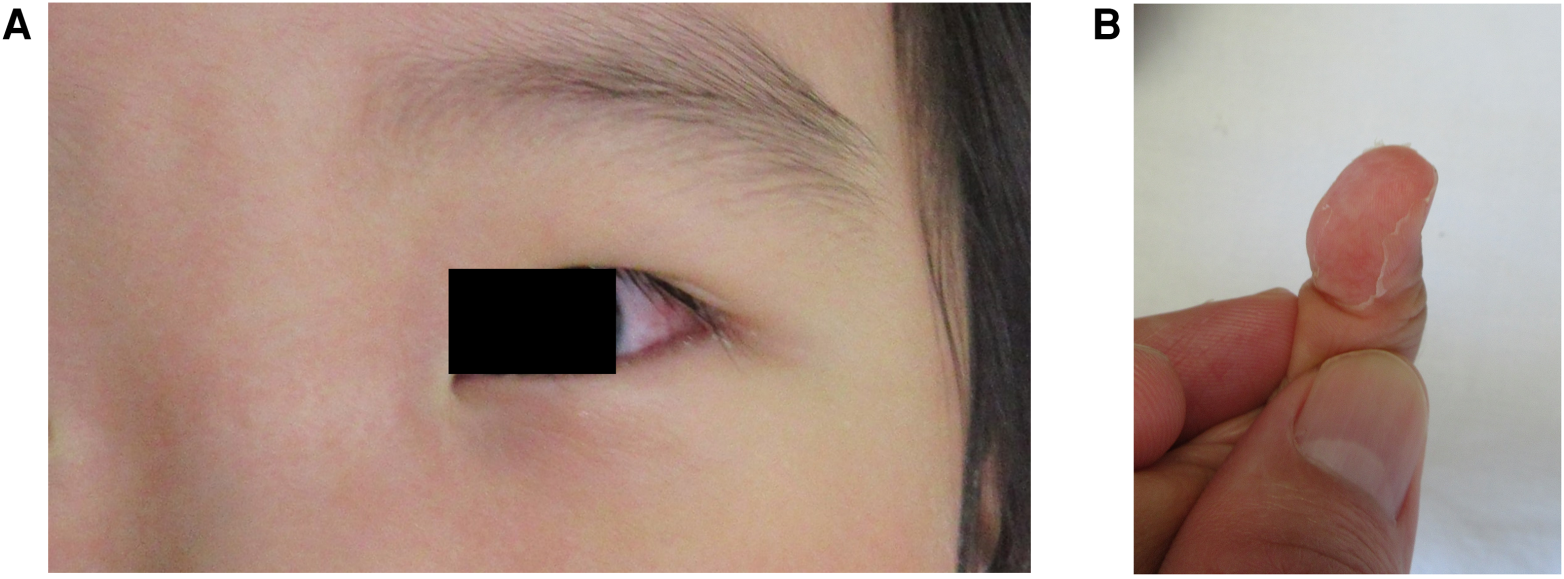
(**A**) Conjunctival hyperemia appeared on day 5 of hospitalization. (**B**) Periungual desquamation on the fingers after treatment.

He developed a second fever on day 5 of hospitalization, despite continuing prednisolone therapy. Also, bulbar conjunctival hyperemia, erythema of the lips, eruption on his trunk, and erythema and edema of the hands and feet were observed (Figure 2A, Supplementary Figures S2A–C). The eruption on the trunk was mainly maculopapular and was not accompanied by blisters, skin detachment, or pustules (Supplementary Figure 2C). No lymphadenopathy or hepatomegaly was observed. Laboratory tests revealed a white blood cell count of 23.4 × 10^9^/L (neutrophils: 81.5%, lymphocytes: 10.0%, monocytes: 6.0%, eosinophils: 2.5%, basophils: 0%), and C-reactive protein of 1.68 mg/dl.

These symptoms met the criteria for KD; therefore, a KD diagnosis was established (5). The patient was administered intravenous immunoglobulin 2 g/kg and oral aspirin.

Consequently, his fever improved on day 7 of hospitalization. Periungual desquamation appeared on day 9 of hospitalization (Figure 2B). The patient was discharged on day 13 without any coronary artery lesions.

A drug-induced lymphocyte stimulation test (DLST) was performed for acetaminophen, cefditoren pivoxil, and carbocysteine to identify the drugs responsible for AGEP. The DLST results for acetaminophen, cefditoren pivoxil, and carbocysteine revealed stimulation indices of 86%, 147%, and 199%, respectively (the cut-off for the stimulation index is >181%). Therefore, carbocysteine was suspected as the cause of AGEP.

## Discussion

This was a rare case of KD following AGEP. The diagnosis of AGEP was established when the patient met the European Study of SCAR group criteria. The DLST suggested that carbocysteine was the suspected cause of AGEP. In this case, it was unclear whether the recurrent fever was associated with AGEP or KD because the symptoms, including fever and rash, occur in both conditions. Because AGEP is a self-limited disease, the recurrent symptoms might be atypical in the course of AGEP. Also, bulbar conjunctival hyperemia was an important finding suggesting that KD sequentially occurred after AGEP.

Skin lesions in patients with KD typically manifest as erythematous rashes, which are most commonly diffuse maculopapular eruptions involving the trunk and extremities, within 5 days of fever onset (3). Urticarial or pustular eruptions are less typical. Two cases of AGEP-like rashes associated with KD have been reported (6, 7). In these previous reports, the rash occurred with other KD symptoms, such as cervical lymphadenopathy and strawberry tongue. Accordingly, a pustular rash similar to AGEP has been documented as a KD symptom. In the present case, the patient's symptoms of fever, pustular rash, and erythema, which are characteristic symptoms of AGEP, were improving prior to the development of KD symptoms, including bulbar conjunctival hyperemia. Approximately 20% of cases with AGEP may exhibit mild mucous membrane involvement at a single site, typically presenting as erosions of the lips (2). The KD guideline by the American Heart Association states that bulbar conjunctival hyperemia begins shortly after fever onset (3). In this case, bulbar conjunctival hyperemia observed during the second febrile episode suggested a possible KD diagnosis. In addition, the DLST results revealed that carbocysteine was suspected to cause AGEP. In this case, the initiation of carbocysteine treatment was 10 days prior to the appearance of the systemic symptoms and exanthematous eruption. The timing of carbocysteine administration also suggested the causal relationship between carbocysteine and the development of AGEP. The appearance of periungual desquamation after globulin therapy confirmed the KD diagnosis. Therefore, the pustular rash was not considered a symptom of KD in this case. KD was suspected to have developed following AGEP.

The pathophysiological mechanism of AGEP is suspected to be a type IV allergic reaction, in which drug-specific T cells are crucial (2). Conversely, the T cell receptor signaling pathway is inhibited in patients with KD (4). The etiologies of AGEP and KD do not coincide. Furthermore, innate immune system anomalies are critical in the development of KD. Hypothesis reports that KD-specific pathogen-associated molecular patterns (PAMPs) and microbe-associated molecular patterns (MAMPs) cause vasculitis, leading to KD (4). PAMPs/MAMPs induce damage-associated molecular patterns (DAMPs), including S100 protein and high-mobility group box 1. PAMPs/MAMPs, together with DAMPs, cooperatively activate endothelial and immune cells, leading to the development of KD. Therefore, microbes or infectious organisms are suspected to trigger the onset of KD. There are some cases of KD associated with skin disorders (8, 9). A case report shows a 17-month-old female with KD following severe sunburn, demonstrating that the blood level of high-mobility group box 1 was elevated in the acute phase and suggesting that the increased DAMPs was associated to the development of KD (8). In this patient, it is possible to hypothesize that DAMPs release from AGEP skin lesions may have contributed to the development of KD.

## Conclusion

In conclusion, we presented a unique case of KD associated with AGEP. Symptoms, including skin lesions and fever, overlap between KD and AGEP. Therefore, differentiating between KD and AGEP is crucial to prevent cardiovascular complications. The development of new symptoms, including bulbar conjunctival hyperemia, and the recurrence of rash despite drug cessation and corticosteroid treatment are valuable distinguishing factors between KD and AGEP.

## Data Availability

The raw data supporting the conclusions of this article will be made available by the authors, without undue reservation.

## Supplementary material

Supplementary Figure S1 Clinical course of the present patient. IVIG, intravenous immunoglobulin; KD, Kawasaki disease.

Supplementary Figure S2 Physical findings on day 5 of hospitalization. (**A**) Erythema of the lips. (**B**) Erythema and edema of the hands and feet. (**C**) Diffuse maculopapular rash on the trunk.
