# Supplementary figures and images for: Case Report: Kawasaki disease associated with acute generalized exanthematous pustulosis secondary to carbocysteine
Source: Front Pediatr. 2024 Mar 22;12:1374448. doi: 10.3389/fped.2024.1374448 (PMC10995226; doi:10.3389/fped.2024.1374448)

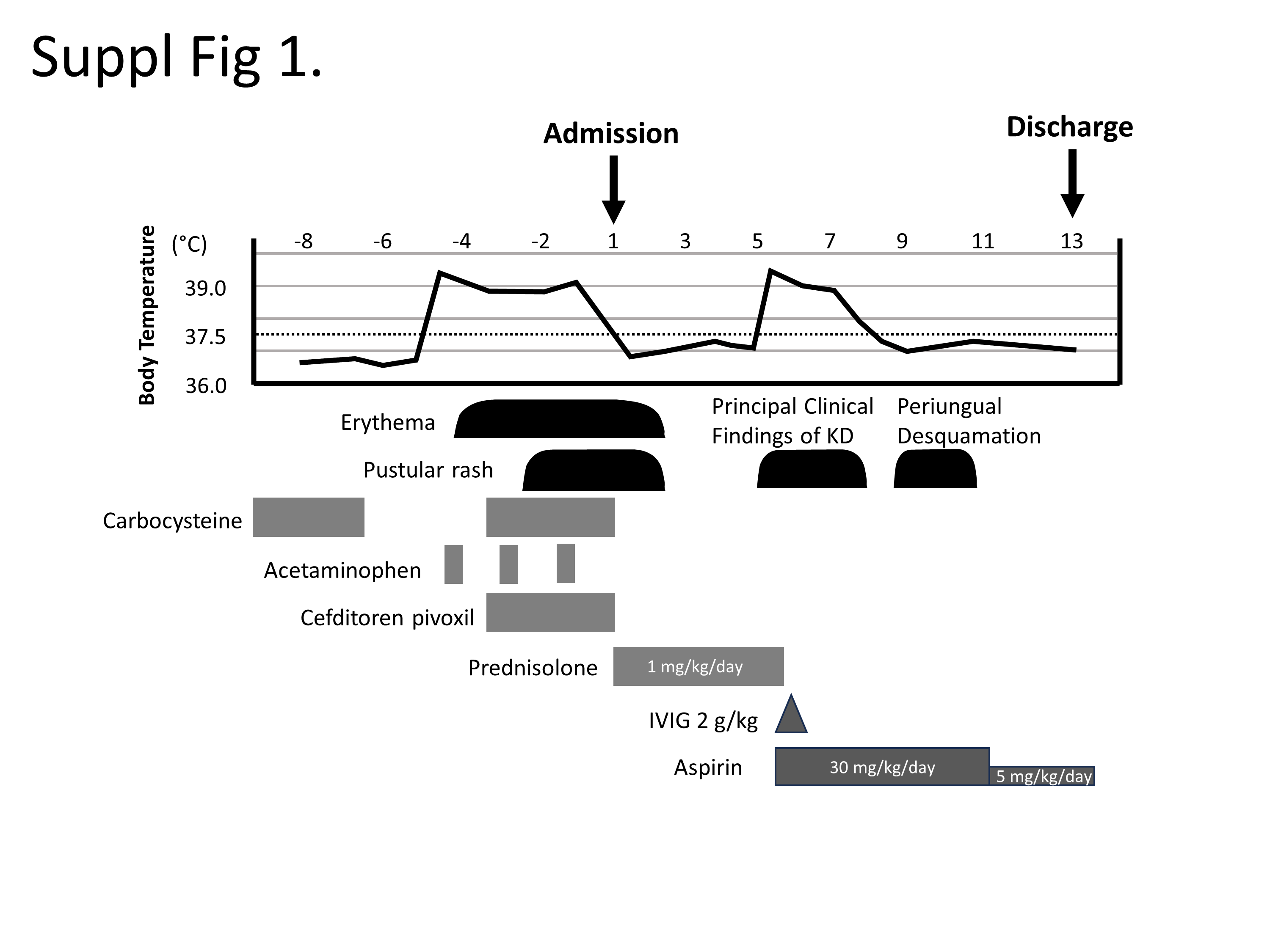

Supplement: Supplementary Figure S1 — Clinical course of the present patient. IVIG, intravenous immunoglobulin; KD, Kawasaki disease. [file Image1.tif]

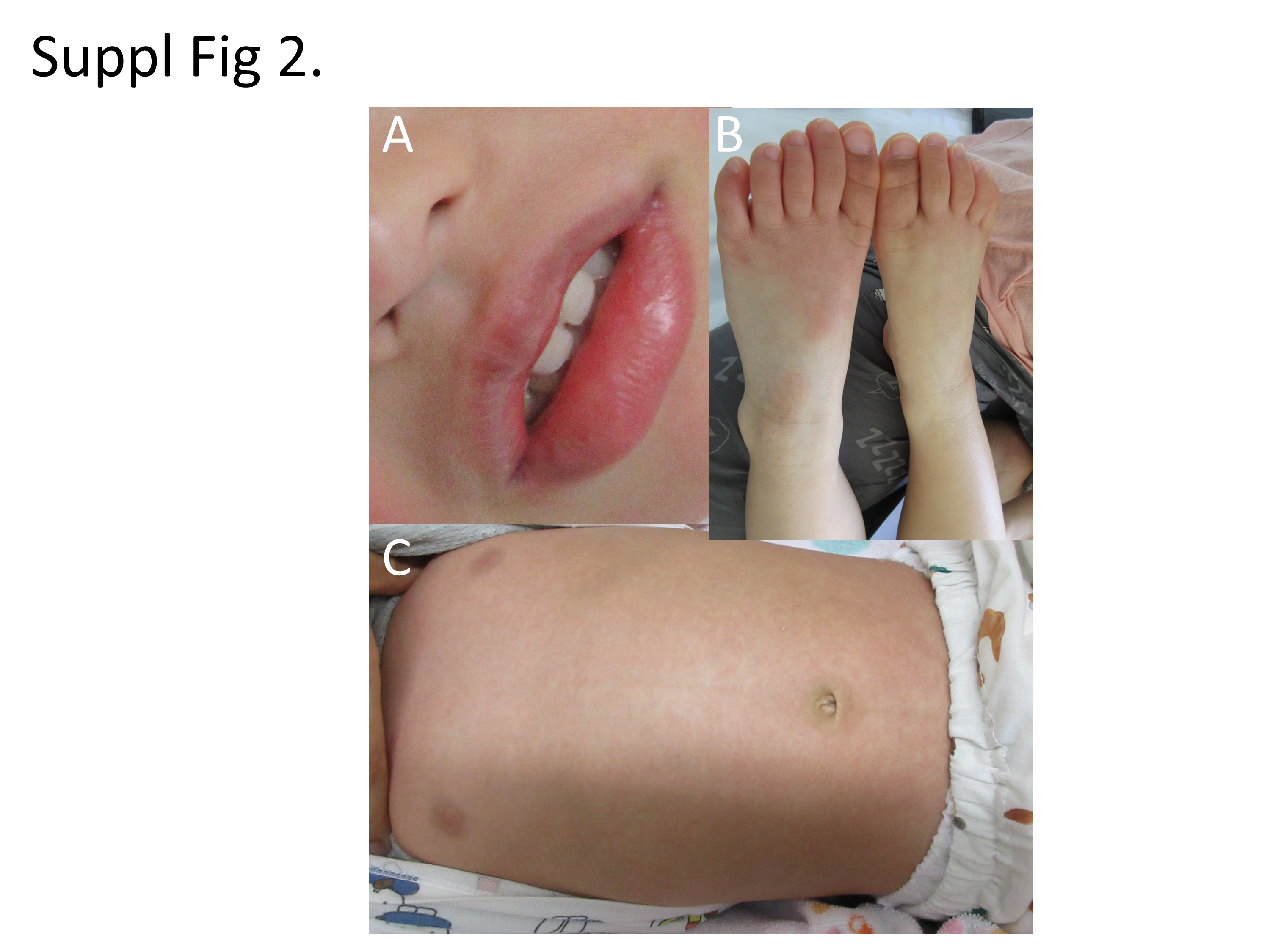

Supplement: Supplementary Figure S2 — Physical findings on day 5 of hospitalization. (A) Erythema of the lips. (B) Erythema and edema of the hands and feet. (C) Diffuse maculopapular rash on the trunk. [file Image2.tif]
